# Adverse Events Following COVID-19 Vaccination in Selected Apartments in Bangalore, India

**DOI:** 10.7759/cureus.21809

**Published:** 2022-02-01

**Authors:** Ananya Chakraborty, Nishith Reval, Latha Kamath

**Affiliations:** 1 Pharmacology and Therapeutics, Vydehi Institute of Medical Sciences and Research Centre, Bangalore, IND

**Keywords:** covaxin, covishield, systemic aefi, local aefi, covid vaccine, aefi

## Abstract

Background

Vaccination has provided a ray of hope in combating the coronavirus disease 2019 (COVID-19). Vaccines were rolled out as an emergency measure, with an expedited approval process. The available clinical trial data reveals the fact that vaccines mostly produce mild adverse events following immunization (AEFIs). Since the experiences are relatively new, it is important to monitor safety in a real-world setting. With this background, this survey was conducted.

Methods

This cross-sectional study was approved by the institutional ethics committee (IEC) of Vydehi Institute of Medical Sciences and Research Centre. This was conducted over a period of four months at select apartment complexes around Whitefield, Bangalore. The participants were invited to fill up data through online Google Forms (Google, Mountain View, CA, USA). They were requested to provide demographic details, information related to vaccination, and AEFIs. Eligibility to participate included recipients of vaccines who received Emergency Use Authorization (EUA) in India. Data were analyzed using SPSS version 20.00 (IBM Corporation, Armonk, NY, USA).

Results

The total number of participants in the study was 322. Out of this, 37.6% (121) were males and 62.4% (201) were females. The mean age of the participants was 34.9 ± 12.4 (mean ± standard deviation (SD) years. About 30% (96) of the study participants had comorbidities. Overall, 67.4% (217) of the participants suffered from AEFI. Of them, immediate reactions were reported by 18.3% (59) and 10.2% (32) of the participants after the first and second doses, respectively. A total of 0.9% (3) of the participants had immediate allergic reactions. The most common local and systemic AEFIs were pain at the injection site and extreme tiredness. AEFIs were found to be mild and with a probable association with vaccination as per the WHO scale. The number of females experiencing AEFIs was found to be higher when compared with males for both local and systemic reactions. There was a statistically significant increase in the number of individuals experiencing general adverse effects following the first dose of Covishield^TM^ (Serum Institute of India Private Limited, Pune, India) when compared with Covaxin^TM^^ ^(Bharat Biotech Limited, Hyderabad, India) (P < 0.05). Of the participants, 5.9% (19) were diagnosed with COVID-19 post-vaccination. Among them, 15.8% (3) required hospitalization, with 10.5% (2) of them requiring an oxygen bed. It was observed that 76.5% (166) of the participants did not report their reactions to concerned authorities.

Conclusion

Based on our sample, the study reflects that COVID-19 vaccination causes mild AEFI in most vaccine recipients. It also provides an insight that reporting of AEFI is very low. It is, therefore, important to take up more awareness campaigns about reporting of AEFIs through the COVID Vaccine Intelligence Network (CoWIN) portal.

## Introduction

Adverse events following immunization (AEFIs) are defined as any untoward medical occurrence that follows immunization and that does not necessarily have a causal relationship with the usage of a vaccine. The adverse event may be any unfavorable or unintended sign, abnormal laboratory finding, symptom, or disease [1]. In India, two coronavirus vaccines received Emergency Use Authorization (EUA) on January 3, 2021. These were Covishield^TM^ (AstraZeneca’s vaccine manufactured by Serum Institute of India Private Limited, Pune, India) and Covaxin^TM ^(Bharat Biotech Limited, Hyderabad, India). The vaccines were in phase III clinical trials [2,3].

Initially, in the first phase, the country vaccinated people at the highest risk of exposure, such as healthcare and frontline workers, on priority with Covishield^TM^, and then, Covaxin^TM^ was made available. From March 2021, the second phase of vaccination was started. Vaccines were available first for people aged above 60 and above 45 with comorbidities. This was expanded on April 1, 2021, to cover everyone above 45 years. In the third phase, vaccines were made available for people above 18 years of age. The vaccination program started at the immunization clinics at hospitals, followed by vaccination drives hosted at outreach sites. The guideline document released by the Government of India mentioned management plans for AEFI and recording of the same through the COVID Vaccine Intelligence Network (CoWIN) software [4].

There were circulars issued by the Pharmacovigilance Program of India (PvPI) to the adverse drug reaction monitoring centers (AMCs) to actively monitor and report coronavirus disease 2019 (COVID-19) vaccine-related AEFIs. The guideline suggested that the AEFIs need to be updated through VigiFlow®. For nonserious reactions, the same should be reported by filling up the suspected Adverse Drug Reaction (ADR) reporting form through VigiFlow®. Serious ones need to be updated through ADR forms to PvPI through VigiFlow®, as well as to the National Coordination Center (NCC)-PvPI by email after filling up the AEFI case notification form. AMCs were also directed to report the cases to the District Immunization Officer or State Expanded Program of Immunization Officer [5-7].

The WHO COVID vaccine safety surveillance manual recommends active and various passive systems to monitor AEFIs. This is to ensure vaccine safety and generate data on the overall short-term and long-term effects [8]. Previous systematic reviews and studies conducted mostly on healthcare workers and published reports on the active surveillance of spontaneous reports by AMCs pointed toward the fact that COVID-19 vaccines are relatively safe. The studies stressed the need for population-based surveillance and a long-term follow-up especially in vaccinated individuals with comorbidities [7,9-15].

With this background, this cross-sectional survey was taken up to assess the self-reported AEFIs of COVID-19 vaccines in select apartment complexes in and around Whitefield, Bangalore, India.

## Materials and methods

It was a cross-sectional, questionnaire-based online survey. It was conducted over a period of four months between July 2021 and October 2021. It was approved by the institutional ethics committee of Vydehi Institute of Medical Sciences and Research Centre, with reference number VIEC/2021/APP/009. Confidentiality was ensured so that only authorized persons had access to the data. The study tool was a Google form questionnaire that was distributed to the residents via emails through the managing committees of the apartment complexes and personal messages through known contacts. The inclusion criteria were vaccinated residents of large apartment complexes who received at least one dose of vaccine that was approved by regulatory authorities in India. The Google form had an introductory section explaining the study and an option to indicate consent. The exclusion criteria included those who received vaccines not approved in India (nonresident Indians) and those who were unaware of the type of vaccine they received. The questionnaire was constructed based on a modified version of the screening questionnaire for adult immunization [16]. The questionnaire included personal information on the respondent’s demographic characteristics, general information related to the COVID-19 vaccine, the type of vaccine received, the number of doses, AEFIs, and health status in terms of medications taken, comorbidity, and details on AEFI reporting. The AEFIs were subjected to causality assessment as per the WHO scale [17].

A pilot survey with 20 participants was first conducted for the purpose of calculating the sample size. It was found that 70% (14) of the vaccinated individuals had AEFIs. Considering the confidence interval at 95%, precision at 5%, and prevalence at 70%, the minimum recommended sample size was derived at 320 (N = Z2pq/d2). It was decided to close the questionnaire link once the desired sample size was achieved.

Baseline data were assessed using descriptive statistics. All quantitative variables are presented as means and standard deviations, and all qualitative variables are presented as frequencies and percentages. For the comparison of categorical variables, the chi-square test was used. Data were entered into Microsoft Excel (Microsoft Corporation, Redmond, WA, USA) and analyzed using SPSS version 20.00 (IBM Corporation, Armonk, NY, USA).

## Results

Demographic characteristics

The total number of participants in the study was 322. Of the 322 participants, 37.6% (121) were males and 62.4% (201) were females. The mean age of the participants was 34.9 ± 12.4 (mean ± SD) years. Of the participants, 3.4% (11) were senior citizens (60 years or above) who participated in the study. Of the participants, 39.8% (128) were between 18 and 30 years, 28.6% (92) were between 31 and 40 years, 23.3% (75) were between 41 and 50 years, and 4.9% (16) were between 51 and 60 years. The mean BMI was 25.2 ± 4.4 (mean ± SD) kg/m^2^. Regarding professional status, 67.7% (218) were non-frontline workers and 32.3% (104) were frontline workers. Of the participants, 96.5% (311) completed both doses, and 3.5% (11) of them completed only one dose of vaccine. The results are presented in Table 1.

Comorbidities

About 30% (96) of the study participants had comorbidities. Around 10% (25) of them were found to have diabetes, 11.2% (36) have hypertension, 3.1% (10) have a thyroid disorder, 0.9% (3) have respiratory disorders, and 0.3% (1) had hypersensitivity reactions to COVID-19 vaccination. The detailed list and frequency of the comorbidities are presented in Table 1.

Type of vaccine

Of the participants, 77.9% (251) received Covishield^TM^ and 22.1% (71) received Covaxin^TM^. The details are shown in Table 1.

Vaccination site

Of the participants, 65.2% (210) received the shot at hospitals, 14.3% (46) at their apartment complex vaccination drives, 13.6% (44) at drives coordinated at workplaces, 0.9% (3) at primary health centers (PHCs), 0.3% (1) at church complexes, and the remaining 5.5% (18) at other sites such as Bruhat Bengaluru Mahanagara Palike (BBMP) office and community halls of residential areas.

Diagnosis of COVID-19 post-vaccination

During the study duration, 5.9% (19) of the participants were diagnosed with COVID-19 post-vaccination. Among them, 15.8% (3) required hospitalization, with 10.5% (2) of them requiring oxygen. The results are shown in Table 1.

**Table 1:** Demographic and vaccination details

Variables	Outcome	Percentage	Frequency
Gender	Male	37.6%	121
	Female	62.4%	201
Age (years)	18–30	39.8%	128
	31–40	28.6%	92
	41–50	23.3%	75
	51–60	5%	16
	>60	3.4%	11
Age (years) (mean ± SD)	34.9 ± 12.4		
BMI (kg/m^2^) (mean ± SD)	25.2 ± 4.4		
Frontline worker	No	67.7%	218
	Yes, I work in the healthcare sector	28%	90
	Yes, I work in a non-healthcare sector	4.3%	14
Comorbidities	Diabetes	10%	25
	Hypertension	11.2%	36
	Thyroid disorder	3.1%	10
	Respiratory disorder	0.9%	3
	Hypersensitivity	0.3%	1
	Psoriasis	0.3%	1
Type of vaccine	Covishield^TM^	77.9%	251
	Covaxin^TM^	22.1%	71
Vaccine site	Hospital	65.2%	210
	Apartment complex drive	14.3%	46
	Workplace	13.7%	44
	BBMP, government setup community halls of residential areas	5.6%	18
	PHC	0.9%	3
	Church complex	0.3%	1
Diagnosis of COVID-19	Post-vaccination	5.9%	19

AEFI

Overall, 67.4% (217) of the participants suffered from AEFI. Of them, immediate reactions within 30 minutes of the vaccine were reported by 18.3% (59) and 10.2% (32) of the participants after the first and second doses, respectively. The most common reactions were extreme tiredness, followed by pain at the injection site. A total of 0.9% (3) of the participants had immediate allergic reactions.

Of the total participants, 63.6% (204) had local reactions following the first dose, and 46.3% (144) had local reactions following the second dose. The most common local reaction was pain, followed by swelling and weakness of the arm. Systemic reactions were reported by 71.4% (230) and 48.5% (151) of the participants, respectively, following the first and second doses. The most common systemic reaction was generalized weakness, followed by fever, headache, chills, dizziness, somnolence, and loss of appetite. The AEFIs included gastrointestinal disorders (nausea, vomiting, decrease in appetite, and pain in the abdomen), nervous system disorders (headache, dizziness, somnolence, and insomnia), skin and subcutaneous tissue disorders (urticaria, hair fall, and rash), musculoskeletal and connective tissue disorders (body pain and pain in extremity), and general and administration site disorders (injection site pain, fever, heaviness, swelling, and tingling). The types of local and systemic reactions with prevalence following the first and second doses are presented in Table 2.

**Table 2:** Details of AEFIs

AEFIs	First dose (N = 322)		Second dose (N = 311)	
	Percentage	Frequency	Percentage	Frequency
Immediate reactions				
Overall	18.3%	59	10.2%	32
Extreme tiredness	11.8%	38	6.4%	20
Pain at the injection site	14.3%	46	6.8%	21
Facial swelling	0.6%	2	0.3%	1
Dizziness/vertigo	0.3%	1	1.6%	5
Generalized severe itching/urticaria	0.3%	1	0.6%	2
Tingling in the left hand and cheek	0.3%	1	-	-
Fever	0.3%	1	2.9%	9
Headache	0.3%	1	-	-
Local reactions				
Overall	63.6%	204	46.3%	144
Pain	59%	190	41.8%	130
Swelling	12.4%	40	6.1%	19
Weakness	9%	29	5.4%	17
Redness	6.2%	20	3.5%	11
Hard lump	5.3%	17	2.9%	9
Fever	0.9%	3	1.6%	5
Heaviness in the injected arm	0.6%	2	0.6%	2
Urticaria/skin problem	0.3%	1	0.3%	1
Koebner phenomenon	0.3%	1	-	-
Systemic reactions				
Overall	71.4%	230	48.5%	151
Feeling tired/generalized weakness	46.6%	150	31.5%	98
Fever	43.4%	140	19.9%	62
Headache	22.4%	72	21.2%	32
Chills	21.7%	70	17.9%	27
Dizziness	8.4%	27	5.9%	9
Somnolence	6.8%	22	7.9%	12
Decrease in appetite	4.7%	15	2.6%	4
Nausea	2.5%	8	2.6%	4
Insomnia	1.9%	6	1.3%	2
Runny nose	1.6%	5	1.3%	2
Cough	1.2%	4	1.3%	2
Vomiting	1.2%	4	0.7%	1
Sore throat	1.2%	4	1.9%	3
Abdominal pain	0.6%	2	1.9%	3
Rash	0.6%	2	0.7%	1
Body pain	0.3%	1	-	-
Leg pain and shoulder pain	0.3%	1	-	-
Discomfort	0.3%	1	-	-
Heavy menstruation	0.3%	1	0.7%	1
Skin problem	0.3%	1	0.7%	1
Severe hair fall	0.3%	1	-	-

Association of AEFI with gender, BMI, and types of vaccine

The number of females experiencing AEFI was found to be higher when compared with males for both local and systemic reactions. The difference was, however, not statistically significant. There was no correlation between BMI and the total number of adverse events (R value = 0.07). The number of participants experiencing local and systemic reactions following the first dose was higher with Covishield^TM^ when compared with Covaxin​^TM^. There was a statistically significant increase in the number of individuals experiencing general adverse effects following the first dose of Covishield^TM^ when compared with Covaxin​​​​​​​^TM^. There was no statistically significant difference in the number of participants experiencing local and systemic reactions following the second dose. The results are shown in Table 3.

**Table 3:** Association of AEFI with gender and type of vaccine * denotes statistically significant difference.

Association of AEFI		AEFI following the first dose			AEFI following the second dose		
Local reactions							
		Yes	No	P value	Yes	No	P value
Gender	Male	70	51	0.11 (X^2^ = 2.53)	48	69	0.15 (X^2^ = 2.53)
	Female	134	67		96	98	
Type of vaccine	Covishield^TM^	170	81	0.06 (X^2^ = 3.45)	111	132	0.17 (X^2^ = ​​​​​​​0.68)
	Covaxin^TM^	34	37		33	35	
Systemic reactions							
Gender	Male	81	40	0.14 (X^2^ = ​​​​​​​2.20)	55	62	0.61 (X^2^ = ​​​​​​​0.26)
	Female	150	51		97	97	
Type of vaccine	Covishield^TM^	186	63	0.02 * (X^2^ = ​​​​​​​5.81)	115	128	0.29 (X^2^ = ​​​​​​​1.14)
	Covaxin^TM^	42	29		37	31	

Pharmacotherapy for AEFI

Of the participants, 71% (154) who experienced AEFI used tablet paracetamol (500/650 mg), and 1.8% (4) used other medications including herbal products, ice packs, antibiotics for hard lump, and mometasone cream. The remaining 27.2% (59) did not require any medication.

Reporting of AEFI and causality analysis

Out of 67.4% (217) of participants who had AEFI, 19.8% (43) reported to the doctor, 3.7% (8) reported to PvPI, and 76.5% (166) mentioned that they did not report their reaction. All AEFIs were analyzed using the WHO-Uppsala Monitoring Centre (UMC) scale. They were found to have a probable association with vaccination. None were serious requiring hospitalization.

## Discussion

In this survey, we aimed to analyze COVID-19 vaccination safety in a select population of Bangalore, India. The participants belonged to apartment complexes located within a 10 km radius of the institution. This was the criteria laid down by the ethics committee, with the idea of monitoring the population who might have received vaccination at the institution and around Whitefield area. This may be also because the adverse drug reaction monitoring center (AMC and PvPI) that functions from the institution supports the nearby peripheral hospitals toward routing their ADR/AEFI to PvPI. The population was targeted toward apartment complexes as request could be forwarded through the MCs, and data could be generated outside the functionality of the hospital. Also, previous studies were conducted mostly on healthcare workers and from AMCs in India [7,10].

The survey was conducted through validated questionnaire uploaded at Google Forms. Participation was voluntary. All information was mandatory; only after responding to a question could the respondents move on to the next. The participants could respond to further questions only if they selected option for vaccine as Covaxin​​^TM^ or Covishield​​​^TM^. This ensured no incomplete responses. It also ensured that the study is conducted on COVID-19 vaccines that received EUA in India. The responses were disabled once the target sample size was reached.

The COVID-19 vaccination drives in India were arranged at various sites. The participants of this study reside in apartment complexes. They received their vaccines at various sites, such as hospitals, apartment complexes, primary health centers, workplaces, community centers, and church complexes. Among the respondents, 37.6% (121) were males and 62.4% (201) were females. The mean age of the participants was 34.9 ± 12.4. The mean BMI was 25.2 ± 4.4 (mean ± SD) kg/m^2^. The majority of the participants were between 18 and 30 years. The results coincide with previous studies where majority participants were females (62.4% versus 37.6%), and females had more AEFI compared with males. Also, among the survey participants, the majority (39.8%) were 20-30 years old, and a smaller number (8.4%) were above 50 years [7,10,11,18]. About 30% (96) of the study participants had comorbidities. In previous studies on AEFI in India, 34.1% of the participants were found to have comorbidities [7]. Also, comorbidities such as diabetes and hypertension topped the list. Our findings coincide with previous studies [7,11,12].

In this study, more participants received Covishield^TM^ (77.9% versus 22.1%). Previous studies also had more participants who received Covishield​​​​​​​^TM^. This may be because of the fact that Covishield​​​​​​​^TM^ was available at many centers in India compared with Covaxin​​​​​​​^TM^ [7]. Of the participants, 5.9% (19) were diagnosed with COVID-19 post-vaccination. Among them, 15.8% (3) required hospitalization, with 10.5% (2) of them requiring oxygen. A survey conducted at Bangladesh found that below 10% (8.5%) of the participants were diagnosed with COVID-19 post-vaccination. Our data thus coincides with the study [12].

In this study, AEFIs were observed more following the first dose of both vaccines compared with the second dose. The results are similar to previous studies [7,10]. Also, in this study, there was a significant increase in the number of participants who experienced general AEFI following the first dose of Covishield​​​​​​​^TM ^as compared with Covaxin^TM^. Till date, there are no comparative studies of AEFIs of these two vaccines. In this study, also, data is based on reporting by participants and in a specific population. Hence, generalized conclusions cannot be drawn. Also, any investigations into the role of active ingredients toward AEFIs are beyond the scope of this study.

Regarding AEFIs, <1% of the participants (0.3%) reported immediate hypersensitivity reaction, and the common localized reactions were pain (59%) and swelling (12.4%). This is similar to previous studies. Also, pain at the site of injection was the most commonly reported adverse event in clinical trials [12,18-20]. The most common systemic reactions were generalized weakness, fever, headache, chills, and dizziness after both first and second doses. Koebner phenomenon was reported by one vaccine recipient. The recipient has a known case of psoriasis. There was appearance of new psoriatic skin lesions at previously unaffected sites due to the cutaneous trauma, and it required medical attention. There are other case reports of skin manifestations after COVID-19 vaccination, including psoriasis flare up. The subjects, however, received their second doses as the reactions were mild and resolved with local medications [21,22].

In the study, the majority of the participants (71%) with AEFIs resorted to tablet paracetamol (500/650 mg) for their symptoms. Out of 67.4% (217) participants who had AEFI, 19.8% (43) reported to the doctor, 3.7% (8) reported to PvPI, and 76.5% (166) mentioned that they did not report their reaction. It is worthwhile to note that none of them reported through CoWIN. Also, the majority of participants did not report their reaction. Our results coincide with a previous study that also detected low reporting and that 74.66% of the subjects were not aware of adverse event reporting [23]. Therefore, it is important to first make vaccine recipients aware of AEFI reporting and encourage them to report through CoWIN. This is required as our experience is very new with COVID-19. AEFIs had a probable association as per WHO scale. This coincides with previous literature [24].

This study was conducted in participants who received the questionnaire and volunteered to respond. Although it was conducted at the community level, we could have possibly skipped those hospitalized or experiencing serious AEFIs. Also, this was conducted on residents of housing complexes. The information that we acquired was based on subjective responses from the population rather than actual clinical assessment. The age range of the respondents was relatively wide, but it did not cover pediatric population as vaccination for COVID-19 was not available below 18 years. Very recently, the Government of India opened up vaccination of children above 15 years. Thus, a long-term study including pediatric population and various sociodemographic profiles of the community will draw more light on long-term AEFIs, if any, of COVID-19 vaccination.

## Conclusions

Our study confirmed the facts available from clinical trial data that available COVID-19 vaccines under EUA are safe. AEFIs were more observed in females, and there were no significant differences in AEFIs between first and second doses and between Covishield^TM^ and Covaxin^TM^.
